# Evaluating the National Rollout of a Type 2 Diabetes Self-Management Intervention: Qualitative Interview Study With Local National Health Service Leads Responsible for Implementation

**DOI:** 10.2196/55546

**Published:** 2024-09-25

**Authors:** Lisa Brunton, Sarah Cotterill, Paul Wilson

**Affiliations:** 1 Division of Population Health, Health Services Research & Primary Care School of Health Sciences University of Manchester Manchester United Kingdom

**Keywords:** type 2 diabetes, structured education, self-management, digital interventions, implementation, qualitative methods, evaluation

## Abstract

**Background:**

Approximately 4.5 million people live with type 2 diabetes mellitus (T2DM) in the United Kingdom. Evidence shows that structured education programs can improve glycemic control and reduce the risk of complications from T2DM, but they have low attendance rates. To widen access to T2DM structured education, National Health Service England commissioned a national rollout of Healthy Living, a digital self-management program.

**Objective:**

The objectives were to understand the barriers and enablers to adopting, implementing, and integrating Healthy Living into existing T2DM care pathways across England.

**Methods:**

We undertook a cross-sectional, qualitative telephone semistructured interview study to address the objectives. In total, 17 local National Health Service leads responsible for implementing Healthy Living across their locality were recruited. We conducted 16 one-time interviews across 16 case sites (1 of the interviews was conducted with 2 local leads from the same case site). Interview data were analyzed using thematic analysis.

**Results:**

Three overarching themes were generated: (1) implementation activities, (2) where Healthy Living fits within existing pathways, and (3) contextual factors affecting implementation. Of the 16 sites, 14 (88%) were implementing Healthy Living; the barrier to not implementing it in 2 case sites was not wanting Healthy Living to compete with their current education provision for T2DM. We identified 6 categories of implementation activities across sites: communication strategies to raise awareness of Healthy Living, developing bespoke local resources to support general practices with referrals, providing financial reimbursement or incentives to general practices, promoting Healthy Living via public events, monitoring implementation across their footprint, and widening access across high-need groups. However, outside early engagement sites, most implementation activities were “light touch,” consisting mainly of one-way communications to raise awareness. Local leads were generally positive about Healthy Living as an additional part of their T2DM structured education programs, but some felt it was more suited to specific patient groups. Barriers to undertaking more prolonged, targeted implementation campaigns included implementation not being mandated, sites not receiving data on uptake across their footprint, and confusion in understanding where Healthy Living fit within existing care pathways.

**Conclusions:**

A passive process of disseminating information about Healthy Living to general practices rather than an active process of implementation occurred across most sites sampled. This study identified that there is a need for clearer communications regarding the type of patients that may benefit from the Healthy Living program, including when it should be offered and whether it should be offered instead of or in addition to other education programs. No sites other than early engagement sites received data to monitor uptake across their footprint. Understanding variability in uptake across practices may have enabled sites to plan targeted referral campaigns in practices that were not using the service.

## Introduction

### Background

Recent figures from Diabetes UK show that approximately 4.5 million people now live with type 2 diabetes mellitus (T2DM) in the United Kingdom [1], a major public health concern. People diagnosed with T2DM are at risk of developing complications that include cardiovascular disease, renal disease, sight problems, and limb amputations [2,3]. The cost of diabetes care accounts for approximately 10% (£8.8 billion [US $11.6 billion] per year and rising) of the total National Health Service (NHS) budget [4]. Most costs are related to managing complications of diabetes [5]; however, when the condition is managed well through taking medications as prescribed and modifying lifestyle factors (such as eating a healthy diet, increasing physical activity, and maintaining a healthy weight), complications can be minimized [1]. A systematic review and meta-analysis identified that group-based education programs were more effective than usual care in improving clinical, lifestyle, and psychosocial outcomes for people with T2DM [6].

In England, the National Institute for Health and Care Excellence recommends offering evidence-based structured education programs to all adults with T2DM (and their family members or carers) at the time of diagnosis as appropriate, with yearly reinforcement and review [7]. T2DM structured education programs such as X-PERT and Diabetes Education and Self-Management for Ongoing and Newly Diagnosed (DESMOND) are usually offered to newly diagnosed patients with T2DM as face-to-face group sessions [8,9]. However, uptake of structured education programs in England is low, with the latest National Diabetes Audit 2021 to 2022 data showing that, of the 65% of newly diagnosed patients with T2DM offered structured education within 12 months of diagnosis, only 7.5% of people attended a session within the first year following diagnosis [10]. Some people choose not to take up diabetes education for reasons such as lack of time due to family or work commitments, disability, or existing multimorbidity or due to associated costs such as traveling to the venue [11]. To widen access to T2DM support in England, the NHS Long Term Plan committed to expanding access to structured education and digital self-management tools, including widening access to Healthy Living for People With Type 2 Diabetes (HeLP Diabetes) [12]. HeLP Diabetes is a self-management, web-based program originally developed and tested in a randomized controlled trial by researchers at University College London (UCL) [13,14]. HeLP Diabetes was shown to significantly (but modestly) reduce blood glucose levels and be cost-effective compared to usual care and considered feasible to deliver at scale [15,16]. The UCL research team iteratively developed an implementation plan to support the implementation of HeLP Diabetes into routine care [17] using normalization process theory (NPT), an implementation theory that is used to explore how new processes become or do not become routine practice [18], as a guide to development. The recommended implementation strategies included engaging with relevant stakeholders in the early stages of implementation, providing educational support materials, conducting general practice site visits and meetings, and conducting audit and feedback to identify how each general practice was performing and providing regular emails and newsletters to services to promote continued engagement [17]. The UCL research team found that the main barrier to implementation centered on the requirement of staff to provide onboarding support, with some nurses feeling that it was not a legitimate part of their role. A main facilitator of successful implementation was providing staff with patient feedback on using HeLP Diabetes, which was considered to motivate staff to continue to engage with the program [17].

Previous literature has identified factors that influence implementation of eHealth technologies into health services [19,20]. A systematic review of reviews highlighted several factors, including the importance of relevant policies and incentives to drive implementation at the organizational level; implementation climate (ie, the fit between the intervention and the organization) and organizational readiness to implement the intervention; individuals’ knowledge and beliefs; and the importance of planning for implementation, including early engagement of key stakeholders, and evaluation to identify system benefits to increase health professional acceptance [19]. A recent narrative review of technology-delivered diabetes self-care interventions identified that the main barriers to implementation included clinicians’ time constraints and concern that interventions may increase workload and patients’ and clinicians’ lack of familiarity with technology or the intervention [20]. The main facilitators to successful implementation included patients’ and clinicians’ perceived value of the intervention and interventions being of low cost to use, deliver, and maintain [20].

### National Rollout of Healthy Living

NHS England commissioned the national rollout of HeLP Diabetes under the name Healthy Living from 2019 onward. An external digital service provider was appointed to develop and deliver the national program. Unlike HeLP Diabetes, the national scale-up of the Healthy Living program is a self-contained and self-directed web-based program with no health professional support beyond initial referral and signposting [21]. NHS England originally planned a phased rollout with 10 early engagement areas expected to adopt Healthy Living first, working with NHS England to develop and test implementation strategies that could be used for national rollout. Early engagement areas were given £30,000 (US $39,375.60) to support implementation efforts. NHS England’s original intention was that the primary referral route would be through general practices. However, in March 2020, due to the COVID-19 pandemic, NHS England amended their implementation plans to (1) enable early engagement areas to delay implementation to address local pressures and (2) enable a self-referral route for people to self-register without invitation from general practices. Implementation across most early engagement areas was delayed until late 2020, and NHS England worked with early engagement areas to amend their original implementation plans as the prioritization of the development of referral routes changed.

National rollout via general practices started in May 2022. National rollout via general practices was to be organized through the 42 integrated care systems (ICSs) across England—ICSs are partnerships of organizations that join to plan and deliver health and care services across a geographic area [22]. NHS England considered 4 implementation options for national rollout (Textbox 1). In response to the multiple pressures placed on primary care following the COVID-19 pandemic, NHS England chose to roll out option 3, whereby Healthy Living was provided to ICSs as an offer of support but with no formal requirements placed upon ICSs to implement the program. NHS England communicated to general practices that Healthy Living was an addition to their existing locally commissioned T2DM structured education programs and should not be considered a replacement for them.

**Implementation options considered for the national rollout of Healthy Living (information received by personal correspondence with National Health Service England).**

**Implementation options**

- Scenario 1: formal, national rollout of Healthy Living that requires that all integrated care systems (ICSs) implement the program
- Scenario 2: formal, partial rollout of Healthy Living that requires only those ICSs that have either none or limited Digital Structured Education provision; other ICSs with Digital Structured Education provision would be able to opt in to implement
- Scenario 3: Healthy Living program as an offer of support—offered to those who want it, but no formal requirement for any ICS to implement it
- Scenario 4: a mix of scenarios 1 to 3

### Aim of Research

Healthy Living is a considerable investment of NHS funds and, although it is based on evidence from the HeLP Diabetes randomized controlled trial [15,16], evidence is required to show its effectiveness now that it has been rolled out nationally. Therefore, researchers from the University of Manchester undertook an independent evaluation of the real-world implementation of Healthy Living, including uptake and engagement, effectiveness and cost-effectiveness, fidelity, and how it is being implemented across sites in England [23]. This part of the research aimed to understand the barriers and enablers to adopting, implementing, and integrating Healthy Living into T2DM care across England.

## Methods

### Design

We undertook a cross-sectional, qualitative semistructured interview design.

### Respondents

Respondents were local leads responsible for implementation of Healthy Living across their locality or ICS.

### Sampling and Recruitment

We aimed to interview at least one participant from each ICS across England (N=42) whether they were currently implementing Healthy Living or not. We received names and work email addresses of potential respondents from the Healthy Living national delivery team at NHS England. Email interview invites, with a participant information sheet attached, were sent to potential respondents, and we sent a reminder email after 2 weeks to nonresponders. We used snowball recruitment, in which additional names were provided by local leads (often in cases in which people had changed job roles). We recruited participants from 16 case sites, and saturation of data was reached as later interviews produced little or no new information or themes.

### Data Collection

We planned to conduct interviews in 2 stages, with local leads in the early engagement areas being interviewed first so that findings could inform subsequent rollout. However, implementation activities in the early engagement areas were delayed due to the COVID-19 pandemic, and so data collection occurred in all sites at 1 time point between August 2022 and December 2022.

Interviews were conducted by the first author, LB, who is an experienced qualitative researcher. Most interviews were conducted over the telephone, but a small number were conducted over Microsoft Teams at the request of the respondents. A topic guide was used to manage the interviews (Multimedia Appendix 1). All interviews were audio recorded on an encrypted digital audio recorder and transcribed verbatim. Transcriptions were subsequently checked for accuracy by LB and anonymized to be ready for analysis.

### Data Analysis

Interview transcripts were uploaded to the NVivo qualitative software (version 12; QSR International) for data management and coding. The data were analyzed thematically [24] using the constant comparative method to systematically interrogate the data [25]. LB read and reread transcripts and created a summary (memo) of each interview to familiarize herself with the data and develop an inductive coding framework. LB coded the interviews, and the coding framework was refined as more interviews were coded. Data were summarized into a table of categories; this was created in Microsoft Word and included selected verbatim quotes to support the categories. We continued to work between the table of categories and the full transcripts and codes in NVivo; this enabled us to generate themes and compare the data systematically to identify the similarities and differences within and across the data. Themes were refined after presenting early findings at 2 wider research team meetings and a patient and public involvement meeting that was attended by 7 members comprising people at risk of developing T2DM, those with a diagnosis of T2DM, and some who cared or had cared for older relatives with T2DM. Throughout the analysis process, LB and PW held regular research meetings to further scrutinize the data and generate the interpretive themes presented. The final set of themes presented was agreed upon by all authors.

### Ethical Considerations

The full research program (of which this study is part) was reviewed and approved by the Yorkshire and the Humber–Leeds West NHS Research Ethics Committee (reference 20/YH/0250). Before the interview started, verbal informed consent was digitally audio recorded using a prepared form that was emailed to respondents ahead of the interview. Data were anonymized during the transcription process to remove any traceable information and maintain participants’ confidentiality. Participants were not paid to take part in the interviews.

## Results

### Respondents

A total of 17 local leads were recruited into the study, and 16 interviews were conducted across 16 sites; 1 (6%) interview was carried out with 2 local leads at the same site. A total of 24% (4/17) of the local leads were from early engagement areas, and 76% (13/17) were from 12 sites that had started to implement Healthy Living following national rollout. The interviews lasted between 23 and 61 minutes (mean 35 minutes).

Most respondents (14/17, 82%) were project or program managers or support officers, but 18% (3/17) of the respondents held more senior strategic, commissioning roles. Most (15/17, 88%) had a remit to work across the whole footprint of their ICS; however, 12% (2/17) of the respondents only worked in a specific locality within their ICS. In total, 41% (7/17) of the local leads had a portfolio of work that involved diabetes alone, whereas most had a broader responsibility for other long-term conditions (8/17, 47%), prevention (1/17, 6%), or commissioning and transformation (1/17, 6%).

We generated three overarching themes from the analysis: (1) implementation activities, (2) where Healthy Living fits within existing pathways, and (3) contextual factors affecting implementation.

### Theme 1: Implementation Activities

#### Overview

A total of 88% (14/16) of the sites were implementing Healthy Living at the time of the interview. Across the 14 sites that were implementing Healthy Living, we identified six categories of implementation activities that occurred: (1) organizing communication strategies to raise awareness of Healthy Living, (2) developing bespoke local resources to support general practices with referrals (in addition to the national resources provided), (3) providing financial reimbursement or incentives to general practices to encourage them to search their registers and send out SMS text messages, (4) promoting Healthy Living alongside other diabetes structured education programs to the local general population via public events, (5) monitoring the implementation of Healthy Living across their footprint, and (6) widening access to Healthy Living across high-need groups. Table 1 outlines each implementation activity reported and which sites used it.

Reasons for not implementing Healthy Living in 12% (2/16) of the sites focused on not wanting to compete with their current structured education provision. In site 1 (early engagement area), patients with T2DM were referred to structured education via a patient referral hub; they did not want Healthy Living to be offered as a first-line program, preferring instead for people to be offered face-to-face services in the first instance. Therefore, they were still trying to work out where to offer Healthy Living within their T2DM care pathway:

And at present in [site 1] [Healthy Living] is yet to be offered to anybody, because we didn’t want to stray away from the actual pathway of [the patient referral hub]...cause the fear would be [if] we offer [Healthy Living] to everyone, and then they would never ever go on to the [patient referral hub]...[that] then stops everybody from having a physical choice of all the other things they could have.

Local lead 03; site 1 (early engagement area)

Site 10 chose to delay implementation of Healthy Living because they had recently commissioned a new digital program and intended to evaluate its effectiveness before they introduced another digital offer into their T2DM care pathway:

[T]he work with [the newly commissioned digital programme] was seen as...let’s not make it more complicated...and then see how we could maybe bring [Healthy Living] into what we’re doing [in future]...there are matters in terms of what are we paying for? And can we see the benefit of that? And this is where doing too much at once would muddy the water.

Local lead 06; site 10

**Table 1:** Implementation activities across case sites.

Implementation activity			Total number of sites undertaking the activity, n (%)		Early engagement areas undertaking the activity, n (%)
**Communications to general practices**			10 (63)		3 (75)
	Include as item in newsletter	7 (44)		2 (50)	
	Direct contact with general practices via email or telephone	4 (25)		2 (50)	
	Disseminate national implementation support resources	4 (25)		2 (50)	
	Webinars undertaken with social prescribing teams and local pharmacy teams	1 (6)		1 (25)	
	Attend local general practice meetings	1 (6)		1 (25)	
	Promote with relevant staff at strategic meetings	6 (38)		2 (50)	
	Promote at local staff educational events	2 (13)		1 (25)	
	Add to general practice email correspondence for T2DM^a^ options	1 (6)		0 (0)	
	Provide specialist teams with email banner to promote Healthy Living	1 (6)		0 (0)	
**Developing bespoke local resources**			6 (38)		2 (50)
	Create search template	1 (6)		1 (25)	
	Create SMS text message template	1 (6)		1 (25)	
	Create flowchart of T2DM structured education offers available (including Healthy Living)	4 (25)		1 (25)	
**Use of financial reimbursement or incentives**			3 (19)		2 (50)
	Provide general practices with direct financial incentive to carry out search of register, send SMS text messages and complete tracker	2 (13)		2 (50)	
	Add Healthy Living to local enhanced service^b^ for T2DM care pathway	2 (13)		1 (25)	
**Raising awareness among the public**			5 (31)		2 (50)
	Promote at local community events	2 (13)		2 (50)	
	Promote via social media channels or request to promote on general practice website	4 (25)		1 (25)	
**Monitoring implementation**			3 (19)		3 (75)
	Request general practices to complete tracker	3 (19)		3 (75)	
	Use data on uptake from NHS^c^ England to monitor implementation performance	3 (19)		3 (75)	
**Widening access**			4 (25)		2 (50)
	Plan to conduct focus groups with people from ethnic minority groups	1 (6)		1 (25)	
	Promote to homeless health care team	1 (6)		1 (25)	
	Promote to local traveler community	1 (6)		1 (25)	
	Translate Healthy Living poster into different languages	1 (6)		1 (25)	
	Plan to directly contact general practices in deprived areas that have not engaged with Healthy Living	2 (13)		2 (50)	
	Request that clinicians signpost patients who decline locally commissioned T2DM structured education to Healthy Living	3 (19)		1 (25)	

^a^T2DM: type 2 diabetes mellitus.

^b^General practices receive financial reimbursement for meeting targets related to T2DM care.

^c^NHS: National Health Service.

#### Subtheme 1.1: Light-Touch Implementation

At the time of data collection, outside the early engagement areas, sites undertook “light touch” implementation activities, including communication strategies to raise awareness of Healthy Living among general practice staff responsible for referring patients to T2DM structured education, discussing the program at local board meetings with those responsible for commissioning T2DM structured education, and raising patient awareness via social media channels:

We haven’t asked people to engage with us...we asked them to circulate to everyone that does the diabetes reviews...offering education is part of the review...so we just want to make sure that everyone that’s carrying out a review is aware of it. And then we also asked them to put it on their websites.

Local lead 08; site 9

I did communicate it out to primary care through our GP bulletin, saying if you would like to promote within your practices, you’re welcome to promote it. So, I haven’t given them all the information the NHS England have given us...so it’s been quite left to primary care if they want to do anything about it.

Local lead 17; site 6

Yeah, we do ongoing comms. I mean, recently, we did a big piece related to the Diabetes Week, as well, so we did some ICS-wide, which is the Integrated Care System, right across.

Local lead 07; site 11

None of the sites from national rollout had been prescriptive regarding how Healthy Living was to be promoted by general practice and other community teams to their populations of people living with T2DM. Some of the early engagement areas reported encouraging general practices to conduct searches of their practice registers to identify eligible people and send out SMS text messages. This was regarded as the method of referral that placed the least burden on primary care given other pressures they faced during and after the COVID-19 pandemic:

I think just sticking to this text messaging method of roll out during the pandemic was the easiest thing for practices to implement. So it, kind of, like broke down some of those barriers of, we don’t have the staff to be printing off letters, or we don’t have the money for postage...it just took away a few of those barriers and helped.

Local lead 1; site 4 (early engagement area)

#### Subtheme 1.2: Monitoring Referrals to Healthy Living

Early engagement areas monitored referrals into Healthy Living across their footprint. This entailed asking general practices to complete a tracker to show how many SMS text messages or letters had been sent out to eligible people; this was cross-referenced with the data received from NHS England to identify how many people had registered or activated an account with Healthy Living to identify the level of uptake. This enabled local leads to consider widening access to Healthy Living to high-need populations. For example, some early engagement areas identified that general practices in deprived areas were less likely to promote Healthy Living to their patients and suggested that more work was needed to raise awareness in these localities, whereas site 4 (early engagement area) had also commissioned a local community group to carry out focus groups as their data showed that people from ethnic minority groups were less likely to register for the program:

So, just looking at the spreadsheet now, those that signed up [to send out text messages to eligible patients]...probably looking at three from that [deprived] area...we need to do more work...and I think it’s across the board in terms of diabetes [care] in general...the uptake from practices and PCNs [primary care networks] [in deprived localities] isn’t as high as other [localities].

Local lead 4; site 5 (early engagement area)

Then we get to...one of our most deprived areas and the highest prevalence of diabetes and only two practices [out of 14] sent text messages...so we need to work there.... In addition, it’s showing low uptake among ethnic minority groups. So, we’ve commissioned a piece of work with a local community group...to do some focus groups and understand why they’re not signing up to Healthy Living.

Local lead 01; site 4 (early engagement area)

None of the sites from national rollout monitored how many people were offered Healthy Living across their area, and at the time of the interviews, they had received no data from NHS England regarding uptake.

#### Subtheme 1.3: How Implementation Monies Were Spent in Early Engagement Areas

As outlined previously, early engagement areas received monies to support their implementation efforts, and these were used in different ways. In one site (site 4), they employed a part-time support officer for 6 months whose main role was to telephone general practices to raise awareness of Healthy Living. They also used the money to commission the focus groups discussed previously. In total, 50% (2/4) of the sites chose to use their implementation monies to provide financial incentives to support general practices to carry out specific clinical register searches for eligible patients and send out SMS text messages. Financial incentives given ranged from £200 (US $262.50) per general practice (site 5) to £1800 (US $2362.54) per general practice (site 7) and were considered a good use of the funds to ensure that general practices engaged with the program in the early adoption phase:

We’ve still got money left over from the pilot still. So, that’s what that was used for...some of them still haven’t applied for the £200. But no, I think it’s a good incentive for them, you know, looking at some spreadsheets that we’ve received back in terms of the trackers, you know, it takes them an hour, and hour and a half at most, so for £200...it’s a good incentive rate for an hour’s work.

Local lead 04; site 5 (early engagement area)

### Theme 2: Where Does Healthy Living Fit Within Existing Pathways?

#### Overview

Most respondents perceived Healthy Living to be an addition to the existing T2DM structured education that they had in place, which was in line with the message from NHS England. Some interpreted this to mean that Healthy Living should be offered to patients only after they had attended (or declined to attend) a locally commissioned face-to-face T2DM structured education program:

And Healthy Living, from a lot of people’s perceptions, including mine, is something that should be offered after a different education has been sought. Because the role of peer support and community [support] has a greater impact.

Local lead 03; site 1 (early engagement area)

The problem we have with local programmes, you can’t make face to face—[people] work...if they can’t access anything locally or haven’t had the opportunity to access anything locally, there is a national programme. So, it is around just best managing their condition and health and wellbeing, et cetera.

Local lead 13; site 14

Local leads expressed mixed views regarding the Healthy Living program. Healthy Living was mainly perceived to be a positive addition to the portfolio of T2DM structured education. Some respondents described how they welcomed a free, national offer that was available to all patients (and their family members or carers) regardless of time since diagnosis of T2DM. This contrasted with their locally commissioned T2DM structured education offers that were often restricted to newly diagnosed patients:

And that’s one of the reasons why I really love, actually, having the universal offer that’s provided by NHS England, because it provides a sort of consistent base...it will just mean that everybody will be able to receive the same information from the same source as a standardised approach...it can be based upon what is needed, rather than what the demand...is for a structured education programme.

Local lead 15; site 16

However, positive views were often tempered, with local leads also expressing reservations regarding where Healthy Living fit within their existing pathways due to the concerns outlined in the following sections.

#### Subtheme 2.1: Duplication and Confusion

Some perceived Healthy Living to duplicate existing locally commissioned digital structured T2DM education services, and despite communication from NHS England that Healthy Living was an addition to and not a replacement for locally commissioned programs, one case site (site 16) reported that they had decided to stop commissioning their local digital offer:

So, with the new offer [Healthy Living] coming out from NHS England, and it being uncapped and available for anybody to access regardless of whether they are newly diagnosed or not, we decided that it would be better to actually focus on that [Healthy Living], rather than advertising another locally commissioned one as well.

Local lead 15; site 16

Others expressed general concern regarding perceived duplication and inefficiency within the NHS:

And this is sometimes the thing with the NHS...you end up with all these offers and all these services and there’s quite often overlap and there’s quite often duplication and it seems like no one quite knows how to best manage it or how to avoid those duplications.

Local lead 02; site 7 (early engagement area)

Moreover, respondents described the numerous lifestyle offers that existed for people with T2DM in primary care and how this led to confusion in understanding what type of patients was suitable for what type of program. Local leads from a number of sites reported that they were still grappling with where Healthy Living fit within their existing T2DM care pathways, which contributed to the “light touch” implementation approach described previously:

I think what we hear a lot from practices, and in fact our colleagues across the system really, it’s the confusion around how many lifestyle programmes are on offer and where to signpost people, where to refer people to. We get that constantly and it is almost no matter how many times you go through the offer, people are still very confused.

Local lead 14; site 15

Question: how do you feel that [Healthy Living] fits with the existing services in your area?

Local lead 10: Well, we found it quite difficult, didn’t we? The reason we took a while to decide to roll it out is we were concerned how does it fit into the structured education on offer? What is the additional requirement?

Site 12

So we’ve tried to not roll it out unless clinicians understood where it might go on their pathway already.

Local lead 09; Site 12

[W]e still get quite a lot of feedback that there is confusion in the system. So if a GP diagnoses someone with diabetes, do they send them to the local structured education or do they suggest they use Healthy Living or do they suggest the other national programme being pushed quite a lot, the digital weight management [programme]? Which is the most appropriate? The GP staff don’t necessarily know. So there’s still quite a lot of confusion around that.

Local lead 11; site 13

#### Subtheme 2.2: Healthy Living Perceived to Be More Suited to Specific Patient Cohorts

Several respondents perceived that the Healthy Living program as a self-directed web-based program without health professional support was geared more toward certain population types; for example, they considered that it may be more suitable for (and appeal more to) native English-language speakers and people who were well educated and motivated to learn, with some suggesting that it would be useful for younger, working-age populations who may not find time to attend a face-to-face course:

As our demographic [with T2DM] are getting younger, they’ll want the app...so I think it will be quite reasonable for a younger person...who are tech savvy to be offered a structured education through an app.

Local lead 16; site 3

I definitely think it’s going to be a good resource for people who are...happy accessing things online and maybe people who have gone through a fair bit of education as well, because you’re used to using this type of resource and reading articles...

Local lead 02; site 7 (early engagement area)

Probably [more suited] to those that are more affluent and digital ready, and people that are of working age and this fits in around their lifestyle, which is the main asset of this programme.

Local lead 03; site 1 (early engagement area)

Local leads perceived it to be less suitable for (and appeal less to) populations who were likely to be more predisposed to T2DM, such as people from specific ethnic minority groups (including those whose first language is not English), people from more deprived areas (including those that were digitally excluded), and people with learning disabilities. This led some to express concern that national programs had the potential to widen health inequalities rather than address them because they perceived such programs as not being tailored toward those with the highest needs. Healthy Living as a web-based, self-directed program was not considered to help ICSs in reaching out to their high-need patients, particularly those who experienced barriers to accessing health care:

But we don’t just want to reach out to the white, the worried well, those that are naturally aware and concerned about their mental and physical wellbeing; I’m not worried about those. Those will naturally reach out...it’s those that don’t and need a bit more support from us to be able to do that. And that’s where I don’t know how suitable Healthy Living would be best placed for that. It’s an open challenge really, but you can say that for any large scale, national programme.

Local lead 12; site 2

And I guess the other thing to consider is the health inequalities aspect. So...a couple of our [localities] have got some quite distinct populations...So it’s actually, how accessible is [Healthy Living] for a lot of our cohorts who are the ones who are most predisposed to having type 2 diabetes? So, it feels as though, even if [Healthy Living]’s there, we’re still going to have to offer something that meets everyone else’s needs.

Local lead 05; site 8

Obviously, I think we’re not solely the only [ICS] that’s raised the concern around digital exclusion. Especially noting that...some of our areas are very deprived and obviously, the cost of living is affecting everybody, so there’s also the potential of the impact from that and the difference in broadband access as well, which should be recognised.

Local lead 09; site 12

### Theme 3: Contextual Factors Affecting Implementation

This theme highlights the contextual factors that impeded sites from conducting more intensive and targeted implementation activities.

#### Subtheme 3.1: Lack of Feedback on Uptake Data

The main contextual factor affecting implementation, and the main barrier to conducting more intensive or targeted implementation activities for local leads from national rollout sites, was not receiving any data from NHS England on uptake. Local leads suggested that it would be useful to receive data on how many patients within their ICSs (or from which general practices) had registered on Healthy Living or how many had activated their accounts or completed the program. Some local leads compared Healthy Living to other nationally commissioned programs such as the NHS Digital Weight Management Programme [26], for which they did receive such data, and questioned why it was not possible to receive similar uptake data for Healthy Living:

So, I think sometimes, having the stats, so when we get the reports from the NHS Digital Weight Management Programme, I think that just gives us a way of communicating [to general practices] how the programme is going. It also gives us an opportunity to put it on as an agenda item as part of our diabetes structured education working group...So I think it would be helpful to hear whether [Healthy Living] is being taken up by people living in [site 15]. Because I wouldn’t really know whether it’s a useful programme for people to access, whether people are accessing it.

Local lead 14; site 15

...there’s only any point in promoting [Healthy Living] if we know that people are actually going to it, completing it, using it and seeing the benefits of it. And I guess the difference for us is, if it’s our own [locally commissioned structured education programme] we get all that information. If we direct them to an NHS England tool, we’ve got no idea whether they turn up or not. But, we’re still measured on it. So there’s a bit of a disconnect for me around, well, if we have no control, how can we influence?

Local lead 05; site 8

While lack of data was not a barrier to initially raising awareness of Healthy Living across their general practices, lack of data *was* a barrier to conducting subsequent, targeted implementation campaigns:

We’re not even going to have any data on how many people are using it for a very long time. So, in the absence of that, it’s quite hard to know what to do...it’s not a barrier to rolling it out, more of a barrier to continuing to push it...

Local lead 08; site 9

What we’ve done is pushed it out, told everybody, said this is what’s available...but we’ve not done any more detailed work with that, and because, we don’t know, do we. So, we don’t know whose picked it up and who hasn’t, so we can’t do that until we’ve got that information.

Local lead 10; site 12

Some respondents also reported confusion in knowing how patients who had registered and attended Healthy Living would be reported back to general practices to enable the latter to code them as attending a structured education program in general practice clinical systems, something they were audited on. This led to concerns that patients who attended Healthy Living may not count in their figures for the number of patients attending T2DM structured education and appeared to be another barrier to implementation. In addition, not receiving data on uptake led to confusion over whether Healthy Living did indeed count as attending a structured education program:

If practices are...not mailing out to all their patients and relying on in-practice advertisements, we won’t be able to map [those patients] to that particular practice because there’s no requirement for an NHS number [to register for Healthy Living]. So, for some patients, there’s no way of knowing who has actually gone on to attend from a particular area, other than just the postcode. So, we’re not going to be able to compare.

Local lead 15; site 16

...an offer of [Healthy Living], would that then meet the requirement of being offered structured education because they’ve got to opt into that themselves and do that self-care themselves...if they get a referral into secondary care [diabetes structured education] it feels more of a must do...as opposed to here’s [Healthy Living] it’s up to you to access it.... I have asked NHS England for our metrics on who’s got access to Healthy Living and haven’t heard anything back, so it’s a bit of a problem.

Local lead 16; site 6

#### Subtheme 3.2: Implementing Healthy Living in a Crowded Market

Another contextual factor affecting implementation related to the perception from local leads that primary care was “bombarded” with communications on the many offers available for patients with T2DM. Local leads were cognizant of not wanting to “overload” general practices and so tread cautiously in the frequency of communications:

Our senior team are having discussions with the primary care leads to find out a better way of doing this so we’re not bombarding them or drip-feeding different things.

Local lead 01; site 4 (early engagement area)

I think it is trying to get it out in a crowded market. In terms of diabetes at the minute...prevention, footcare, low calorie, there’s a lot of stuff going off at the minute anyway.

Local lead 04; site 5 (early engagement area)

#### Subtheme 3.3: Timing of National Rollout

Early engagement sites were faced with implementing the program just as the COVID-19 pandemic happened, and this severely delayed their implementation. For sites that implemented Healthy Living following national rollout, local leads described how primary care was still dealing with COVID-19 recovery efforts and continued to be overstretched due to winter pressures and organizing influenza and COVID-19 vaccination programs. Given that there was no formal mandate for ICSs to implement Healthy Living, local leads were reluctant to request additional activities such as searches of patient registers or sending out SMS text messages to eligible people:

We could definitely do more around the promotion of NHS Healthy Living programme [but] we always play that fine line...we don’t want to overwhelm primary care any more because they are under a lot of pressure. They have been under pressure through the pandemic, they are under the winter pressures now with COVID and flu vaccination programmes.

Local lead 14; site 15

When you talk to PCN managers or the practices themselves, they’ve got a lot going on and when you try and talk to them about DESMOND...there’s potentially still a drawback...because they’ve got other, you know, the winter pressures, the COVID...so then also trying to fit in or send them resources about Healthy Living...it’s like “well, we haven’t got capacity at the moment to do that.”

Local lead 04; site 5 (early engagement area)

National rollout occurred in May 2022, which was also a time of organizational change for the NHS as ICSs were in the process of being formed. Some local leads described how their focus was on conducting scoping exercises to identify diabetes offers across localities within their ICSs and working toward creating a more consistent and equitable offer of T2DM services across their localities. This suggested a lack of capacity to concentrate efforts on implementing Healthy Living beyond conducting light-touch implementation activities:

And then we are also doing lots of work around diabetes recovery at the moment. We are doing lots around recovery of the 8 care processes and the three treatment targets. So, trying to recover back from pre-pandemic levels.

Local lead 14; site 15

There was a sense among local leads that the national rollout of Healthy Living happened with little fanfare in comparison to other national programs, and they felt that regional NHS England leads had not been proactive in providing information about Healthy Living:

I’m not swimming in knowledge around Healthy Living, nor has it felt like there’s been a tap on the shoulder around why are you not [implementing it].

Local lead 06; site 10

## Discussion

### Principal Findings

Most sites were implementing Healthy Living across their ICS, but this was limited to raising awareness of Healthy Living via one-way communications to general practices. Thus, a more passive process of dissemination (as opposed to active implementation) was used by most sites. Early engagement areas received funds from NHS England to support implementation, and these were used to conduct more intensive implementation activities, with 50% (2/4) of the early engagement areas choosing to provide general practices with financial incentives for undertaking referral tasks. Local leads were generally positive about Healthy Living as an additional offer to existing service provision, although concern was raised that national programs such as Healthy Living had the potential to increase health inequalities rather than widen access to structured education because they were felt not to be geared toward patient cohorts with the greatest need. While most early engagement areas were monitoring uptake of Healthy Living across their area and making attempts to widen access to Healthy Living, no sites implementing Healthy Living following national rollout monitored general practice referrals. Contextual factors such as the absence of data received on the uptake of the program, no formal requirements being placed on ICSs to implement the program, and the timing of national rollout impeded more prolonged, targeted implementation activities to support rollout of Healthy Living across most sites.

### Limitations

We initially aimed to analyze the interviews through the lens of NPT [18] as a more mandated and active implementation process was anticipated. However, expectations placed upon sites to implement Healthy Living changed from what we initially envisaged when we designed the study. In addition, the national rollout of the Healthy Living program removed the onboarding support provided by general practice clinicians in the HeLP Diabetes service, thereby removing any direct patient engagement with health professionals in the Healthy Living program [21]. Therefore, general practice implementation efforts were reduced to signposting eligible patients to Healthy Living so that patients could self-register for the program. As NPT is focused on the concept of “collective action,” or what people do to implement new practices and ways of working, its use was less relevant to a process of passive dissemination [17]. Instead, we deployed a thematic analysis approach using the constant comparative method to systematically interrogate the data.

We aimed to recruit at least one local lead from every ICS in England (N=42) but recruited a total of 17 local leads across 16 different sites (16/42, 38% response rate). Therefore, the findings may be limited by nonresponder or “sampling bias,” that is, the underrepresentation of sections of the population under study [27] Gaining views from a convenience sample of those who agreed to the interviews may reflect different views from those of sites that did not take part. Nevertheless, sites varied in terms of geographical areas of England, including rural and urban locations, and included sites that had adopted Healthy Living in the early rollout phase, those that had implemented Healthy Living following national rollout in May 2022, and those yet to implement the program. In addition, our sample of 16 ICSs was spread evenly across the range of deprivation seen among all ICSs in England [28], with 6 (38%) in the most deprived areas, 4 (25%) in the middle, and 6 (38%) in the least deprived areas. The proportion of the population that was of people of color ethnicity in our sample of 16 ICSs ranged from 3% to 54%, with a mean of 17%. This is the same as the national mean for the proportion of non-White populations among all ICSs [29]. Hence, we are satisfied that the sample provided an adequate mix of sites to evaluate the implementation of Healthy Living. In addition, while the number of interviews was lower than anticipated, we are satisfied that, in rigorously collecting and analyzing interview data, we were able to represent the “full dimensionality of [participant]’s experiences” [30]. A recent systematic review confirmed that qualitative studies can reach data saturation with sample sizes between 9 and 17 interviews [31].

Findings are also limited by the cross-sectional design as we undertook interviews at 1 time point; therefore, interviews report a “snapshot” in time. It would be useful for future evaluations to consider conducting interviews across different time points to investigate changes in implementation over time.

### Comparison With Prior Work

We identified that the main barrier to prolonged or targeted implementation campaigns was the absence of data on uptake, which meant that local leads often did not go beyond raising awareness of Healthy Living across their localities. An updated systematic review of systematic reviews on factors that influence the implementation of eHealth technologies recommended that implementation should not stop with “go-live” and identified the need for continued monitoring and evaluation of eHealth technologies to ensure that intended goals were being met, benefits were realized, and barriers to use were understood [19].

Another barrier to implementation was confusion in understanding where Healthy Living fit within existing T2DM care pathways, with some indicating that Healthy Living should be offered to people only after attending or declining to attend a locally commissioned face-to-face T2DM structured education program. In contrast to local leads’ views, qualitative research from the Healthy Living Diabetes-Long-Term Independent National Evaluation study exploring patients’ experiences of using Healthy Living [32] identified that participants felt Healthy Living to be most suitable for newly diagnosed patients, perceiving the program to provide a reliable source of introductory information about T2DM. Some suggested that Healthy Living could be offered alongside face-to-face programs, which provide additional health care professional and peer support. In addition, most participants in the study had taken part in structured education programs but still learned new information from Healthy Living. They especially valued the emotional management content in Healthy Living as they had not been made aware of the emotional impact of T2DM previously, including when attending face-to-face T2DM structured education courses [32].

Research from the Healthier You NHS Diabetes Prevention Programme (DPP) showed that patients registered with practices identified as providing lower-quality care were less likely to be referred to the NHS DPP, leaving such patients disadvantaged, concluding that using primary care for referral into the NHS DPP had the potential to further increase health inequalities [33]. This study highlights that sites struggled to engage general practices in the most deprived areas to undertake referrals to Healthy Living and that some of the early engagement areas were planning future engagement work with those practices but there were no similar plans in other sites. This highlights the importance of monitoring where referrals are being initiated from and providing support to practices with lower referral rates [33].

We found evidence that money allocated to the implementation of Healthy Living in the early engagement areas was used, in whole or in part, to provide general practices with “process based” financial incentives (ie, payments received for undertaking referral tasks). Payment for performance, or the use of financial incentives, in health care systems worldwide is increasingly used to try to improve quality and efficiency [34]. Previous research suggests that outcome-based incentives (ie, payments directly linked to referral volumes) are effective in increasing referrals but process incentives, such as those used in early engagement areas, are not [35].

### Implications for Future Practice and Policy

Healthy Living is a self-contained, self-management digital health intervention without support for onboarding, health coaches, or any health professional support. Local leads responsible for implementing Healthy Living across their area perceived Healthy Living to be more suitable for patients who were self-motivated to learn and those who were more highly educated. This perception contributed to them not carrying out targeted implementation campaigns for Healthy Living. Healthy Living was implemented into an already “crowded market,” with a number of other local and national programs competing for attention (such as locally commissioned T2DM structured education or lifestyle programs, the Digital Weight Management Programme [26], and the Path to Remission program [36]). There is a need for clearer communications regarding the type of patients who may benefit from the Healthy Living program, when it should be offered, and whether it should be offered instead of or in addition to other programs.

As reported, none of the sites other than early engagement areas received data to monitor uptake across their ICSs. Understanding variability in uptake between practices may have allowed sites to plan targeted referral campaigns in those practices that were not using the service.

### Conclusions

This study describes the impact of shifting from an active to passive strategy for the implementation of Healthy Living. Passive dissemination, whereby communications were sent to general practices to raise awareness of Healthy Living, were favored over more active implementation processes. This was due mainly to wider contextual factors but the strategy of awareness raising, while necessary, is unlikely to drive uptake of and engagement with the program in general practice, if used alone.

## Appendix

Multimedia Appendix 1 Topic guide.

## Abbreviations

DPP: Diabetes Prevention Programme
DESMOND: Diabetes Education and Self-Management for Ongoing and Newly Diagnosed
HeLP Diabetes: Healthy Living for People With Type 2 Diabetes
ICS: integrated care system
NHS: National Health Service
NPT: normalization process theory
T2DM: type 2 diabetes mellitus
UCL: University College London

## Data Availability

The data sets generated during and analyzed during this study are available from the corresponding author on reasonable request.
