# Supplementary material for: Evaluating the National Rollout of a Type 2 Diabetes Self-Management Intervention: Qualitative Interview Study With Local National Health Service Leads Responsible for Implementation
Source: J Med Internet Res. 2024 Sep 25;26:e55546. doi: 10.2196/55546 (PMC11464934; doi:10.2196/55546)
Supplement: Multimedia Appendix 1 [file jmir_v26i1e55546_app1.doc]

**Before interview starts:**

- Explanation of the objective of interview
- Explanation of ethics, consent and confidentiality of interview and analysis
- Structure and duration of the interview
- Any questions?
- Record verbal consent

**Background:**

1. **Ask them to explain their professional background/current role**
2. **What current provisions do they have in place for diabetes education programmes (including digital)?**
3. **Are they are implementing ‘Healthy Living’ in their area?**
4. **If no, what are reasons behind choosing not to implement the programme?**
5. **Probes:**
   1. Concerned that it would displace current diabetes provision/ or is not required?
      1. How does ‘Healthy-Living’ fit with existing local service provision for type 2 diabetes management?
      2. How is it different to existing local service provision?
   2. Do they have challenges in being able to resource implementing ‘Healthy Living’ into primary care?
6. **If yes, what is their role in relation to establishing the local ‘Healthy-Living’ online self-management programme**?
   1. Establish if they are an early engagement area

1. **Explore how local leads perceive ‘Healthy living’ might make a difference to the management of patients with type 2 diabetes, how it fits with existing services in their area**

Probes:

- 1. What do they understand to be the aims of the ‘Healthy-Living’ self-management online programme?
  2. What sorts of benefits do they expect from implementing ‘Healthy-Living’ within their area?
     1. Explore whom they perceive to be the target population
  3. What sort of challenges did they expect from implementing ‘Healthy-Living’ in their area?
     1. Were these challenges realised and if so, what were they and how did they overcome them? (this may be covered in 10.a – only probe here if it is timely to do so)
  4. How does ‘Healthy-Living’ fit with existing local service provision for type 2 diabetes management?
  5. How is it different to existing local service provision?
  6. Has it or do they think it might displace current diabetes education provision that is currently funded?

1. **Explore the ‘engagement work’ done to support implementation of ‘Healthy-Living’ across their area**

Probes:

1. Explore what activity is happening across their area
   1. who is involved in helping to promote awareness to patients/refer patients? (may not just be general practice) – ask if we could speak to these people for phase 2b.
2. Explore the rationale provided to CCG leads/ primary care/community re: implementing ‘Healthy-Living’?
3. How have they promoted/raised awareness of ‘Healthy-Living’ programme to CCG leads/Practices?
4. Do they feel CCG leads/practice staff or other community/primary care staff engage willingly with ‘Healthy-Living’?
   1. Willingness of others to support the implementation of ‘Healthy-Living’ (e.g. CCG leads promoting service and clinicians tasked with referring patients onto programme)
   2. Degree of openness in primary care/community to work in new ways
   3. Are there differences across their area?
5. **Experience of implementation within existing type 2 diabetes care pathways**

Probes:

1. How do they think ‘Healthy-living’ has impacted ways of working in primary care/community?
2. How are referral and clinical pathways being embedded?
3. Are resources available to support primary care/community in referring patients into ‘Healthy-Living’?
   - Do they provide practices / community services with resources? (e.g. reimbursement to conduct patient searches/for postage or texts etc.) Where do these resources come from?
4. Do they have any concerns?
5. **Explore how respondents evaluate whether ‘Healthy-Living’ is being implemented effectively within their area; suggestions for future improvement**

Probes:

- 1. What challenges have they faced in implementing ‘Healthy-Living’ in their area?
     1. How have they overcome challenges?
  2. What went well in implementing ‘Healthy-Living’ in their area?
     1. Explore why they think this is
  3. Have they experienced any intended or unintended consequences from integrating ‘Healthy-Living’ into existing type 2 diabetes care pathways at an area level?
  4. Any suggestions about future development of the ‘Healthy-Living’ service?
  5. What are their views on the sustainability of the ‘Healthy-Living’ service?
     1. Can they identify any barriers to long-term sustainability?
     2. Can they identify any facilitators to long-term sustainability?

1. **What advice would they offer to other areas that are about to implement ‘Healthy-Living’?**
2. **Do they have anything to ask or say before we finish interview?**
